# Subsets of CD1c^+^ DCs: Dendritic Cell Versus Monocyte Lineage

**DOI:** 10.3389/fimmu.2020.559166

**Published:** 2020-09-30

**Authors:** Lukas Heger, Thomas P. Hofer, Venetia Bigley, I. Jolanda M. de Vries, Marc Dalod, Diana Dudziak, Loems Ziegler-Heitbrock

**Affiliations:** ^1^ Laboratory of Dendritic Cell Biology, Department of Dermatology, Friedrich-Alexander University of Erlangen-Nürnberg (FAU), University Hospital Erlangen, Erlangen, Germany; ^2^ Immunoanalytics-Tissue Control of Immunocytes and Core Facility, Helmholtz Centre Munich, Munich, Germany; ^3^ Translational and Clinical Research Institute, Newcastle University, Newcastle upon Tyne, United Kingdom; ^4^ Department of Tumor Immunology, Radboud Institute for Molecular Life Sciences, Radboudumc, Nijmegen, Netherlands; ^5^ Department of Medical Oncology, Radboud Institute for Molecular Life Sciences, Radboudumc, Nijmegen, Netherlands; ^6^ Aix Marseille Univ, CNRS, INSERM, CIML, Centre d’Immunologie de Marseille-Luminy, Marseille, France; ^7^ Deutsches Zentrum Immuntherapie (DZI), Erlangen, Germany; ^8^ Comprehensive Cancer Center Erlangen-European Metropolitan Area of Nuremberg (CCC ER-EMN), Erlangen, Germany; ^9^ Medical Immunology Campus Erlangen, Erlangen, Germany; ^10^ Monocytomics Research, Munich, Germany

**Keywords:** DC2, CD1c, CD172, CD301, CD14, dendritic cells, DC subsets

## Abstract

Currently three bona fide dendritic cell (DC) types are distinguished in human blood. Herein we focus on type 2 DCs (DC2s) and compare the three defining markers CD1c, CD172, and CD301. When using CD1c to define DC2s, a CD14^+^ and a CD14^−^ subset can be detected. The CD14^+^ subset shares features with monocytes, and this includes substantially higher expression levels for CD64, CD115, CD163, and S100A8/9. We review the current knowledge of these CD1c^+^CD14^+^ cells as compared to the CD1c^+^CD14^−^ cells with respect to phenotype, function, transcriptomics, and ontogeny. Here, we discuss informative mutations, which suggest that two populations have different developmental requirements. In addition, we cover subsets of CD11c^+^CD8^−^ DC2s in the mouse, where CLEC12A^+^ESAM^low^ cells, as compared to the CLEC12A^−^ESAM^high^ subset, also express higher levels of monocyte-associated markers CD14, CD3, and CD115. Finally, we summarize, for both man and mouse, the data on lower antigen presentation and higher cytokine production in the monocyte-marker expressing DC2 subset, which demonstrate that the DC2 subsets are also functionally distinct.

## Introduction

In human blood, cells with dendritic cell (DC) properties have been classified as plasmacytoid DCs (pDCs), as CD141^+^ DCs and as CD1c^+^ DCs (1–3). CD141^+^ DCs are also termed DC1s or cDC1s, while CD1c^+^ DCs are defined as DC2s or cDC2s, with “c” standing variously for conventional or classical (4).

The pDCs express CD123 and CD303 and are characterized by their unique ability to produce high amounts of type I Interferon (5).

The CD141^+^ DCs co-express CD370 (CLEC9A) (6–8) and they are described to activate CD8^+^ T cells *via* the MHC class I pathway including cross-presentation of exogenous antigen to CD8^+^ T cells (9–11). Their high ability to cross-present antigen from necrotic cells may be due to the expression of CLEC9A, since this receptor was shown to efficiently bind necrotic cells (12) *via* binding to actin filaments (13).

CD1c^+^ DCs can present antigen to both CD4^+^ and to CD8^+^ T cells (9, 14), however, when cultured with necrotic cells then they are inferior to CD141^+^ DCs in cross-presentation of necrotic cell derived antigen (9). The CD1c^+^ DCs form the largest DC subset in human lympho-hematopoietic tissues (8). Due to their efficacy in antigen presentation and T cell activation, CD1c^+^ as well as CD141^+^ DCs are attractive cell populations for vaccination studies with primary blood DCs (15, 16).

For all of these three DC types, at least two subsets have been described: for the pDCs a CD2^−^ and a CD2^+^ subset has been reported (17), for CD141^+^ DC there is a XCR1^−^ and a XCR1^+^ subset with the XCR1^−^ cells being the putative precursors of the XCR1^+^ DCs (18). Finally, within the CD1c^+^ DC population a differential expression of CD5 and of the monocyte-associated CD14 molecule has been reported. The CD14^+^ subset shows higher expression levels for several additional monocyte associated markers. This prompts the question whether the CD14^+^ and CD14^−^ subsets have a different ontogeny and specifically whether the CD1c^+^ CD14^+^ cells are linked to the monocyte lineage. With a focus on man and mouse, these questions will be addressed herein.

### Markers to Define DC2 Cells

The initial question is, whether there are reliable markers in man and mouse to define DC2s as compared to CD141^+^ DCs and to monocytes/macrophages. There are three markers used for DC2s and these are i) CD1c, ii) SIRPα (CD172a) and iii) CLEC10A (MGL or CD301). For the purpose of this review, we will preferentially use the CD nomenclature.

**CD1c** is a frequently employed marker for DCs in man (1). CD1c is part of the MHC-like CD1 family of genes and it is involved in the presentation of lipid-based antigens to T cells (19). Importantly, while CD1c is found in many species including horses and panda bears, no murine homologue could be identified.

In human blood, CD1c was consistently found to label a population distinct from CD141^+^ DCs and from classical monocytes (20). In addition, CD1c expression is strongly expressed on almost all B cells (21), making it important to exclude CD19^+^CD20^+^ B cells when defining CD1c^+^ DCs. Moreover, it had been noted early on that CD1c, even after exclusion of B cells, is not restricted to DCs since it can be induced readily on monocytes by culture with GM-CSF within one day (22). Also, CD1c can be found on CD141^+^ DCs after FLT3L injection into apparently healthy volunteers (23). Of note, even CD141^+^ cells isolated from human skin appeared to co-express CD1c (24). Taken together, although the marker CD1c is widely used for the description of the DC2 subset, one should be aware of the fact that the molecule is not uniquely expressed on the DC2s, when performing flow cytometry or immunohistological analyses.

**CD172a (SIRP-α)** is another marker frequently used to define DC2s. CD172a is a transmembrane glycoprotein, consisting of three extracellular Ig-domains and two intracellular ITIM motifs that mediate negative signals after binding of CD47 to the N-terminal Ig-domain (25).

In man, CD172a is expressed by blood and tissue CD1c^+^ cells but it is low on CD141^+^ DC1s in various tissues (26). However, CD172a is also expressed by granulocytes and by monocytes (27) and this is also the case in pigs (28). Therefore, several additional markers are needed for unequivocal identification of DC2 cells in blood and tissue when using CD172a.

Similar to man, CD172a also selectively stains mouse DC2s but not DC1s. In the mouse spleen, CD172a is strongly expressed by lineage-negative CD11c^+^CD4^+^ but not by CD11c^+^CD8^+^ DCs (29), which is consistent with a selective staining of DC2s. Also, in mouse thymus a CD11c^+^CD8^+^SIRPα^−^ and a CD11c^+^ CD11b^+^CD8^−^SIRPα^+^ (=CD172a^+^) cell population was described, and these represent the DC1 and DC2 subsets, respectively (30, 31). Others found, however, that CD172a is not completely absent from CD103^+^ DC1 cells, since in ocular mucosa it is expressed at a low level by these cells (32). Still, it was suggested that mouse CD172a^+^ DC2s can be clearly separated from CD172a^−/low^ DC1s when the latter are defined *via* XCR1 (33). In conclusion, CD172a is a suitable marker for the definition of DC2s, but as several other cell types express this marker, these cells need to be carefully excluded in flow cytometry analyses.

**CD301 (CLEC10A, MGL**) has been suggested more recently as a defining marker for human CD1c^+^ cells (8, 34). CD301 is identical to MGL (macrophage C-type galactose/*N*-acetylgalactosamine-specific lectin) and it acts as an endocytic receptor. In the mouse, it was cloned from elicited peritoneal cells (35) and the human gene was cloned from monocytes after 7-day culture with IL-2 (36). CD301 is expressed by monocytes cultured with GM-CSF and IL-4 for 7d (37). Also, very low levels of CD301 mRNA and protein were reported for intermediate monocytes (38). Hence, there is an apparent association of CD301 with monocytes/macrophages.

In this context, Heger et al. (34) have assessed CD301/CLEC10A for its suitability as a marker for DC2s. In these studies, CD301 was highly selective for CD1c^+^ DCs. Only a small fraction of thymic B cells and a subset of monocytes/macrophages in the spleen was found positive for CD301 under steady-state conditions (34). Therefore, CD301 appears to have great potential as a unique marker for DC2s in man, with only some expression on monocytes/macrophages to be considered.

In the mouse, CD301 exists in two forms with different carbohydrate specificities, namely MGL1 and MGL2 (39). Based on structure, expression pattern, and carbohydrate specificity, mouse MGL2 (also termed CD301b) appears to be the homolog of human MGL (CLEC10A, CD301) (40). Staining of bone marrow cells with anti-MGL antibodies identifies a population of cells that is solely positive for MGL1 and another population positive for both MGL1 and MGL2. Hence, antibodies against MGL1 stain more cells, and this includes pDCs and macrophages. Also, anti-MGL2 stains peritoneal macrophages (40). Additional studies using a Mgl2-DTR/GFP DTR-cell-depleting mouse model suggest a role of MHC class II^+^ CD11c^+^CD301b^+^ cells in resistance against HSV2, and these cells were suggested to be DCs (41). Further studies reported on CD11c^+^CD301b^+^ cells, which were addressed as DCs (42), while F4/80^+^CD11b^+^CD206^+^CD301b^+^ were defined as macrophages (43). Also, CD301b^+^CD11c^+^CD11b^+^MHCclassII^high^F4/80^int^CD206^+^ mononuclear phagocytes were described in various tissues including fat, liver, and muscle with very few cells seen in blood (44). In addition, Langerhans cells in the mouse skin show a strong signal for MGL (45) but it still needs to be determined whether this is CD301a or CD301b. Taken together, CD301 in the human system and CD301b in the mouse system are promising identifiers of DC2s, but additional markers and a careful approach are required for correct identification of these cells.

### Summary Statement on DC2 Markers

While in the past, CD1c has been the main marker to define human DC2s, it may well be that CD172a and CD301 might serve a similar function. Comparative analysis may be helpful to define, which of these markers or which combination thereof is most appropriate to define DC2 cells.

In summary, the three markers that can be used to define DC2 cells (CD1c, CD172a/SIRPα, and CD301/CLEC10A/MGL) are not exclusively expressed by these cells. Therefore, they need to be combined with additional markers to exclude B cells, pDCs, and monocytes/macrophages, as appropriate.

### DC2 Markers in Inflammation

While the expression profiles of CD1c, CD172a, and CD301 apply to homeostasis, additional markers may have to be added in inflammatory disease where cytokines can induce DC2-associated markers on other cell types. For example, as mentioned earlier CD1c can be found on CD141^+^ DCs after FLT3L injection into apparently healthy volunteers (23). With the singular use of CD1c as a DC2 marker, such FLT3L-induced cells would be wrongly assigned to the DC2 lineage.

Since monocytes are CD172a-positive in the steady state and since *in vitro* culture of monocytes with GM-CSF can induce expression of CD1c (22) and of CD301 (37) it is important to exclude monocytes/macrophages, when defining DC2 cells in blood and more importantly in tissue. This is particularly relevant in the context of inflammatory diseases when cytokines like GM-CSF are increased (46). An informative example is sickle cell disease, which goes along with increased blood GM-CSF levels (47), with increased numbers of CD16^+^ monocytes (48) and with expression of CD1c on monocytes (49). It remains to be determined whether these monocytes in the blood of sickle cell disease patients are akin to the CD14^+^ subset of CD1c^+^ DCs. In any event, these cells may contribute to the pathophysiology of the disease *via* production of inflammatory cytokines.

Overall, these deliberations show that determination of DC2s in inflammatory conditions requires additional steps in order to unequivocally define these cells.

## Characterization of Subsets of DC2 in Man

### DC2 Subsets in Human Blood

In the 2010 nomenclature proposal it had been noted for the CD1c^+^ myeloid DCs in human blood that these DCs can be separated into CD14^−^ and CD14^low^ cells (1). This was based on the original studies by Thomas and Lipsky (50) demonstrating antigen presenting activity in a population of CD33^++^CD16^−^CD14^+^ cells, cells that later were shown to be CD1c^+^ (51). Of note, the typical approach to DC analysis in human blood starts with the exclusion of lymphocytes and of monocytes. Monocytes are excluded using a CD14 antibody, but depending on the reagent and the conditions used, this may or may not remove CD14^low^ cells.

**Figure 1:**
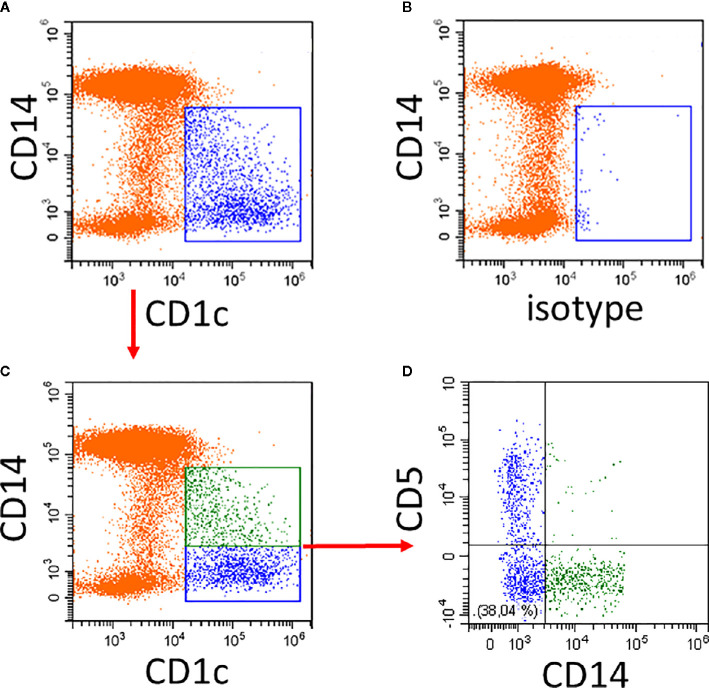
Illustration of CD1c^+^ DCs and its subsets in human blood. Whole blood was stained with CD14, CD16, CD19, HLA-DR, and CD1c antibodies and the expression of CD1c **(A)** compared to isotype control **(B)** was analyzed on HLA-DR^+^ non-B cells. Of note, the CD14^low^ CD1c^−^ cells in **(A)** represent the CD16^+^ monocytes. **(C, D)** show additional staining for CD5. In the example in **(C)**, the CD1c^+^ cells are divided into a CD14^+^ subset (green) and a CD14^−^ subset (blue). As shown in **(D)**, the CD14^−^ subset in blue can be further subdivided into CD5^+^ and CD5^−^ cells. In the lower left there is a population of CD14^−^ CD5^−^ cells. Red arrows indicate the gating sequence.

An example of the CD14 expression pattern of CD1c^+^ DCs is given in **Figure 1**. Compared to the isotype control, there is strong CD1c-staining among the CD14^−^ cells and a gradually decreasing expression of CD1c among the CD14^low^ cells (**Figure 1A**). The isotype control shows a few events within the CD1c^+^ DC2 gate (**Figure 1B**).

The cells within the CD1c gate can then be separated into CD1c^+^CD14^+^ and CD1c^+^CD14^−^ cells (**Figure 1C**) with the CD14^+^ cells (green in **Figure 1A**) accounting for about 40% of all CD1c^+^ DCs. The CD5^+^ cells are distinct from the CD14^+^ cells.

Recently, in a study not excluding CD14^+^ cells, it was reported that in apparently healthy donors about one third of the CD19^−^CD1c^+^ cells are CD14^+^ (15). These CD1c^+^CD14^+^ cells, compared to the CD1c^+^CD14^−^ subset, were shown to express similar levels of HLA-DR and CD33 but higher levels of CD11b and clearly higher levels of PD-L1 (CD274) (15). Upon LPS stimulation, these cells showed a trend to produce higher amounts of TNF and IL-10, but they were less efficient in inducing T cell proliferation induced by allogeneic leukocytes. The T cell proliferation could be improved by addition of an anti-PD-L1 antibody (15). Furthermore, while the CD1c^+^CD14^−^ subset readily induced IFNγ production in CD4^+^ T cells, the CD1c^+^CD14^+^ subset completely failed to do so. Only when CD1c^+^CD14^+^ DCs were stimulated with GM-CSF or LPS then a low level of IFNγ production could be induced. This suggests that the CD1c^+^CD14^+^ do not induce but rather impede T cell proliferation and differentiation toward the TH1 lineage.

In blood of melanoma patients with metastatic disease, the frequency of the CD14^+^ subset of CD1c^+^ cells in blood was found to be increased more than three-fold. Upon vaccination with antigen-loaded CD1c^+^ DCs, patients with a high proportion of the CD1c^+^CD14^+^ subset showed lower T cell proliferation to control antigen (15). This underscores the notion that CD1c^+^CD14^+^ cells in cancer patients are not potent T cell stimulators but rather show suppressive activity. Together, these findings were taken to design an optimized cellular vaccine, in which the CD14^+^ subset is removed from the CD1c^+^ DC product for vaccination of patients with melanoma and other malignancies (15, 52).

Transcriptome analyses comparing the CD1c^+^CD14^+^ cells to CD1c^+^CD14^−^ cells showed the CD1c^+^CD14^+^ cells to express higher mRNA levels for MafB and the CSF1-receptor (CD115) and lower levels for FLT3 and IRF4. In addition, higher levels for TLR7, TLR8, CLEC7A (CD369), CLEC12A (CD371), and CLEC12B were found for the CD1c^+^CD14^+^ DCs (15).

**Figure 2:**
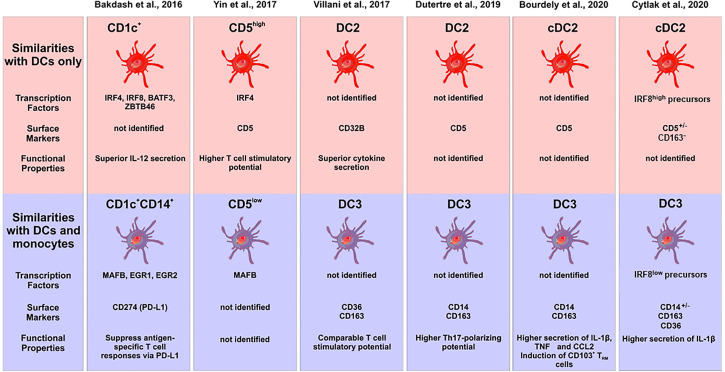
Characterization of subsets of human DC2s in the recent literature. The different studies are listed at the top, the upper panel gives the subsets with pure DC features, the lower panel shows the subsets with monocyte features. Characteristic transcription factors, cell surface markers and functional properties are given when available. The cellular images are provided and adapted from Servier Medical Art (smart.servier.com).

Hierarchical clustering using these transcriptome data suggested that the CD1c^+^CD14^+^ DC2 subset to be in between classical monocytes and the CD1c^+^CD14^−^ cells but closer to the classical monocytes (15). However, a comparison to a comprehensive set of blood DCs and monocytes is still required in order to assign them to either monocytes or DCs, when using transcriptomics as a tool. The central features of DC2 subsets in this study by Bakdash et al. are summarized in **Figure 2**.

The existence of two subsets of DC2 in man was confirmed recently in single cell sequencing studies on peripheral blood mononuclear cells (53). Here, both subsets were positive for CD1c, CLEC10A and FcϵR1A. One subset expressed higher transcript levels for MHC class II molecules and CD1c, while the other was higher for S100A8/9, ANXA1, F13A, VCAN (versican), FCN1 (ficolin 1), RNase2, CD163, and CD14. Many of the latter molecules are associated with monocytes but both CD1c^+^ subsets clustered separately from monocytes in this study. This work by Villani et al. is also listed in **Figure 2**.

Furthermore, Schroder et al. have studied the properties of cells isolated with CD1c-magnetic beads from human blood mononuclear cells, and they noted a CD1c^+^CD14^−^ population and a CD1c^+^CD14^+^ population, with the latter showing low level CD1c (54). Here, a higher expression of CD135 (FLT3) on the CD1c^+^CD14^−^ cells and a higher expression of CD115 (M-CSFR) on the CD1c^+^CD14^+^ cells was observed, and the CD1c^+^CD14^+^ subset was interpreted to represent monocytes.

In early studies, a differential expression of CD5 had been reported on human blood DCs (55, 56). More recently in a 2017 study, the lineage-negative HLA-DR^+^CD123^−^CD11c^+^CD1c^+^ cells have been subdivided into CD5^low^ and CD5^high^ cells (57). Gene expression analysis showed higher SIGLEC6 and IRF4 transcripts in the CD1c^+^CD5^high^ cells, while the CD1c^+^CD5^low^ cells expressed higher levels of CD14, MAFB, S100A8/9, RNAse2, CD163, and Ficolin1. A few of these transcripts were tested at the protein level and here the higher expression of CD14 and S100A9 was confirmed for the CD1c^+^CD5^low^ cells (57). This work by Yin et al. is listed in **Figure 2**.

The reciprocal gene expression pattern for CD1c^+^CD5^high^ cells (57) and the CD1c^+^CD14^low^ cells (15) suggests that these two subsets might be mutually exclusive and that CD1c^+^ cells might consist of CD14^−^CD5^high^ and CD14^+^CD5^low^ cells. As illustrated in **Figure 1**, CD5 and CD14 are expressed on distinct cell subsets (see **Figure 1D**).

This pattern is consistent with what has been described by Meyerson et al. (58). The latter study and our illustrative figure demonstrate a population of CD1c^+^CD5^−^CD14^−^ cells and the question is, whether this subset represents a distinct population. In this context, Dutertre et al. (59) analyzed DCs and their subsets with an extensive panel of cell surface markers. In this study, more than 300 protein markers were employed and markers HLA-DQ and FcϵRIA on DCs and CD88 and CD89 on monocytes were identified as best discriminating markers. On this basis, the DC2s including the CD14^+^ subset could be phenotypically separated from classical monocytes, albeit there is low level expression of both CD88 and CD89 on DC2s.

DCs are thought to be specifically governed by FLT3 (Fms-Like Tyrosine Kinase 3) and injection of this growth factor into patients was shown to result in a shift of the proportions of DC2 versus classical monocytes. Here, both CD1c^+^CD14^+^ and CD1c^+^CD14^−^ DC2s increased relative to the classical monocytes arguing for a DC nature of the CD1c^+^CD14^+^ cells (59). A more detailed analysis in the same report then revealed four distinct subsets of CD1c^+^ cells, which are one CD5^+^ subset and three CD5^−^ subsets, the latter consisting of CD14^−^CD163^−^, CD14^−^CD163^+^ and CD14^+^CD163^+^ cells. Some salient features of the typical DC2 and the subset with monocyte features is given in **Figure 2** (see Dutertre et al).

Three different phenotypes of DC2 cells in human blood were also described in a 38-marker CYTOF analysis (60). The DC2s showed differential expression levels for CD172a and CD163 and the authors concluded that there were CD172^high^CD163^low^ and CD172^low^CD163^med^ and CD172^high^CD163^high^ DCs. The relationship of these three phenotypes to the CD14^+^ und CD5^+^ subsets remains to be determined.

Taken together, among the CD1c^+^ DCs there is higher expression for several monocyte-associated genes (CD14, CD115, MAFB, S100A8/9, CD163, and Ficolin1) in cells defined either as CD14^+^ cells or as CD5^−^.

### Lineage Assignment of DC2 Subsets

In order to appropriately address the question of lineage assignment of the subsets of CD1c^+^ DCs, approaches using a broad panel of different monocytes, macrophages, and dendritic cells are required.

In a recent report on human CD1c^+^ DC2 subsets, the CD5^+^ cells were defined as cDC2, while cells positive for CD14 and CD163 were termed DC3 (61). The CD14^+^ subset of CD1c^+^ cells, when compared to the CD1c^+^CD5^+^ DCs, was shown to express higher levels of TNF and CCL2 and to induce features of tissue-resident memory cells in CD8^+^ T cells (see summary in **Figure 2**). Furthermore, it was demonstrated that GM-CSF but not FLT3L is able to support development of the CD1c^+^CD14^+^ DCs in a humanized mouse model. In *in vitro* studies, GM-CSF was able to induce these cells from a granulocyte-monocyte-dendritic cells precursor (GMDP) but not from common dendritic cell precursor or cMoP, indicating that this subset may have a distinct developmental pathway (61).

In addition, functional studies might be able to address the question of lineage assignment. To this end, recently the ability of DCs to activate the inflammasome and induce the release of IL-1β has been revisited (62) and it was shown that among the GM-CSF induced mouse bone marrow-derived cells only the macrophages but not the DCs were efficient producers of IL-1β. If a relevant IL-1β production by the CD14^+^ subset of DC2 can be demonstrated then this would add another monocyte characteristic to these cells. Cytlak et al. addressed this question by comparing human CD1c^+^CD5^+/−^CD163^−^ DC2, and CD1c^+^CD163^+^ DCs (termed DC3) (63). Here, it was noted that the CD1c^+^CD163^+^ cells, when stimulated with a mixture of TLR ligands followed by intracellular staining and flow cytometry, showed IL-1β production as high as monocytes, while the CD1c^+^CD163^-^ cells showed a low level production of this cytokine (see summary in **Figure 2**). Similar results were obtained for IL-10, while the two DC subsets produced comparable amounts of IL-12 (63).

Moreover, DC2s can be generated *in vitro* from CD34^+^ hematopoietic stem cells (64) and more specifically from cells with the phenotype of MLPs, CMPs, and GMPs (65). The generation of subsets of DC2 was only studied recently (63). Here, CMPs and CD33^+^ GMPs were found to have CD1c^+^CD14^+^ DC2 potential and the CD14^+^ subset was shown to segregate with monocytes. In contrast, CD1c^+^CD14^-^ DC2s could be generated from CD123^+^ GMPs and segregated with pDC and cDC1 potential.

Mutations of genes involved in development of DCs can be informative as to lineage assignment. Homozygous and heterozygous loss of function and dominant negative mutations of the *IRF8* gene have been described. It was shown that bi-allelic loss of function mutations of the *IRF8* gene led to a complete absence of DCs and monocytes (66, 67).

For two independent cases with recurrent disseminated BCG infection and heterozygous dominant negative mutation in the *IRF8* gene, normal numbers of monocyte subsets and no decrease of pDCs and DC1s were initially reported, while there was an apparent reduction in CD1c expression on CD11c^+^ cells (66). In a later study by the same researchers, in a more detailed analysis using CLEC9A in addition to CD141 for DC1 definition and CD1c for DC2 identification, it was noted that DC1s are, in fact, decreased and the decrease of CD1c expression was confirmed (68).

Cytlak et al. looked at CD1c^+^ DCs in a kindred with a heterozygous dominant-negative mutation of *IRF8*, which led to a moderate deficiency of this transcription factor (63). This went along with depletion of cDC1 and pDC but increased blood monocytes and normal numbers of CD1c^+^ DC2s. Analysis of subsets, however, revealed an almost complete absence of the CD1c^+^CD5^+^ cells, while the CD1c^+^CD5^−^ subset was expanded (63). Whereas the moderate level activity of IRF8 is apparently sufficient to allow for development of the CD14^+^ monocyte subsets and of the CD1c^+^CD14^+^ DC subset, it is not sufficient to allow for generation of CD1c^+^CD14^−^CD5^+^ DCs. This indicates that the CD14^+^ and CD5^+^ subsets of CD1c^+^DCs have distinct developmental requirements and that the CD1c^+^CD14^+^ DC2 subset is associated with monocytes. Analysis of additional mutations of genes involved in DC development in man may help to further support this point.

When looking at IRF8 CRISPR/Cas9 knock-out mutation in human *in vitro* induced pluripotent stem cells (iPS), it was noted that the generation of pDC and DC1 cells driven by FLT3L, SCF, GM-CSF, and IL-4 (FSG4) was ablated but the generation of CD1c^+^ DC2s was unaffected (69). These data indicate that this intriguing *in vitro* system more closely mimics partial IRF8 deficiency *in vivo*. Still, manipulation of the IRF8 gene penetrance may help in unravelling the *in vitro* development of DC2s and their subsets. On the other hand, the generation of DC2s in the absence of IRF8 in this *in vitro* system may be due to the strong effects of the exogenous cytokines, since without lineage specifying cytokines, DC2 cells are absent in these IRF8^−/−^ cultures similar to what is seen in immuno-deficient patients (69). Again, it will be important to analyze DC2 subsets in this system.

Moreover, Borriello et al. reported on the expression of the receptor for thymic stromal lymphopoietin (TSLPR), which is induced by stimulation *via* TLR4 in CD1c^+^CD14^low^ cells among human blood mononuclear cells (70). TSLP directs type-2 immune reactions and it acts on a broad range of leukocytes including T cells, macrophages, DCs, mast cells, and ILCs indicating that its receptor is widely expressed (71). Borriello et al. noted that induction is specific to the CD1c^+^CD14^low^ cells and that there is little induction in CD16^+^ monocytes. It will be important to assess the induction of the TSLPR in the CD14^−^ subsets of CD1c^+^ DC2s, in order to determine whether expression might be specific to the monocyte-related subset.

Taken together, while in the studies by Borriello et al. and Sontag et al. (69, 70) the analysis of DC2 subsets is still outstanding, the data on development of DC2 subsets show evidence of co-segregation of the monocyte-marker expressing DC2 subset with monocytes.

### DC2 Subsets in Human Tissue

Because of the ease of accessibility, many human studies are performed on blood samples, but there are also a number of studies that look at DCs in human lymphoid and non-lymphoid tissues (8, 24, 60, 72–75) but only a few studies address subsets of DC2s in tissue.

Of note, interpretation of data in human tissue has to be done with caution: While in blood the monocyte subsets can be clearly defined and dissected from DCs, it is more difficult in tissues to exclude macrophages that may have up-regulated DC2-associated markers. Furthermore, such DC2-marker-positive macrophages may or may not co-exist with bona fide monocyte-lineage derived cells, and this can make lineage-assignment very demanding.

With respect to subsets of DC2s Yin et al. noted CD5^+^ and CD5^−^ subsets of CD1c^+^ DCs in human tonsils and similar to blood the CD5^+^ cells formed the minor subset (57).

Looking at single cell suspensions isolated from the nasal mucosa, all of the CD1c^+^ cells expressed low levels of CD14 (76). Another study on nasal, bronchial, and intestinal mucosa showed low-level CD1c expression on the CD11c^high^ subset of CD14^+^ cells (54).

In the human lung, CD14^+^ and CD14^−^ subsets of CD1c were observed in lavage samples and transcriptome analysis demonstrated higher ZBTB46, FLT3, CD83, and CCR7 mRNA levels in the CD14^−^ subset, while the CD1c^+^CD14^+^ subset showed higher CD36, CD163, CD369 (=CLEC7A=Dectin-1), and S100A8/9 (77). Hence, the CD14^+^ subset of CD1c^+^ cells in the lung alveoli enriched for monocyte-associated genes. Also, in an analysis of bronchoalveolar lavage samples from healthy volunteers, a BTLA^+^ and a BTLA^−^ subset of CD1c^+^CD11c^+^ cells were noted and here a higher expression of monocyte/macrophage-associated genes CD14, CD163, S100A8, and CD115 was noted at the transcript level for the BTLA^−^ cells (78). For the human intestine, Watchmaker et al. identified among the lineage-negative, HLA-DR^+^CD11c^+^ cells two subsets of CD172a^+^ cells, one CD103^+^ and one CD103^−^. Hierarchical clustering using transcriptome data demonstrated that the CD103^−^CD172^+^ cells clustered with blood monocytes and the authors suggested that they might be monocyte-derived (72).

In summary, there is evidence for a monocyte-marker positive DC2 subset in various human tissues like tonsil, lung, and intestine.

## Characterization of Subsets of DC2 in the Mouse

### Differential ESAM Expression for Definition of Mouse DC2 Subsets

For the mouse similar to man, pDCs, DC1s, and DC2s have been described (79–82). However, data on DCs in mouse blood are scarce, and most studies are done on tissue samples. Regarding subsets of DC2, CD11c^+^CD8^−^ cells in mouse spleen and mesenteric lymphnodes Kasahara and Clark described subsets of DCIR2^+^DCAL2^−^ and DCIR2^−^DCAL2^+^, i.e. CLEC4A4^+^CLEC12A^−^ and CLEC4A4^−^CLEC12A^+^, respectively (83). Here, the CLEC4A4^−^CLEC12A^+^ cells exclusively produced TNF and IL12 in response to the TLR9 ligand CpG. Separate studies by Lewis et al. on cells from mouse spleen and intestine revealed that DC2-type cells with the phenotype CD8^−^CD11c^++^CD11b^+^ cells can be subdivided into an ESAM^high^ and an ESAM^low^ subset (ESAM = Endothelial cell–selective adhesion molecule) (84). Here, the ESAM^high^ cells expressed higher levels of MHC class II molecules, while the ESAM^low^ cells showed higher transcript levels for M-CSFR, CCR2, Lysozyme, CD14 and CD36, markers which are typical of the monocyte lineage. Furthermore, the ESAM^low^ subset was able to produce higher levels of TNF and IL-12, when stimulated with the TLR-9 ligand CpG DNA. Also, activation *via* TLR-2, using heat-killed Listeria monocytogenes, led to superior TNF production by ESAM^low^ cells. Phagocytosis of latex beads was similar for both subsets as was the capacity to present *in vitro* OVA peptide using transgenic responder T cells expressing OVA-specific TCR (OT-II cells) (84). However, when testing antigen presentation *in vivo* a robust response of OT-II cells was noted in wild type animals, while in knock-out mice, lacking ESAM^high^ cells, only a minimal response of OT-II cells was observed. This suggests that the ESAM^high^ cells are required for an efficient antigen presentation *in vivo* (84).

Importantly, the ESAM^low^ cells were noted to be CLEC12A^high^ (84) thereby linking the studies by Lewis et al. (84) and Kasahara et al. (83). The conclusion from both studies is that the ESAM^low^CLEC12A^high^ subset of CD11c^+^CD8^−^ mouse DC2s expresses monocyte-associated markers and is more potent in producing cytokines TNF and IL-12.

Also, Lewis et al. (84) demonstrated that blockade of Notch2 signalling led to selective ablation of the ESAM^high^ subset. This ESAM^high^ subset was shown to derive from DC precursors, while the ESAM^low^ cells were suggested to be myeloid progenitor-derived. In other words, Notch2 signalling is required for the development of ESAM^high^ DC2s but not for ESAM^low^ DC2s (84). In an *in vitro* study, DCs were generated from progenitor cell lines by culture with a FLT3L and Notch ligand expressing cell line. Here, transcriptome analysis revealed that with Notch2 activation the CD11b^+^CD24^−^CD8^−^ DC2s were similar to the splenic ESAM^high^ DC2 subset (85).

Mutations of human Notch2 have been described in two families with the patients showing multiple abnormalities of liver, lung, heart, and kidneys and typical facial features (86). At this point, no immunological workup has been published on such patients, and it is therefore not known whether these patients have abnormalities of the immune system.

As mentioned above, a bi-allelic mutation of the IRF8 gene leads to a depletion of DC2 cells in man (66). By contrast, in the mouse it was shown that *Icsbp* (=*Irf8*)^−/−^ animals lack pDC and CD8^+^ DCs (DC1), while CD4^+^ DCs (DC2) were readily detected (87, 88). No information on subsets of DC2s is available, and it remains to be determined whether there is a differential effect on ESAM^high^ versus ESAM^low^ subsets in in *Irf8* ^−/−^ animals.

Taken together, in the mouse the ESAM expression level can be used to define subsets of DC2 with ESAM^low^ subsets showing features of monocytes.

### T-Bet Expressing DC2s

Another recent study by Brown et al. defined in mouse lymphoid organs two subsets of lin^−^CD90^−^CD64^−^Ly6C^−^CD11c^+^ MHCII^+^XCR1^−^CD11b^+^cDC2s based on their differential expression of the transcription factor T-bet (89). The T-bet^+^ DC2s, dubbed DC2A, were found to overlap with the ESAM^+^ DC2s previously described by Lewis et al. (84). The T-bet^−^ cDC2s, called DC2B, showed higher expression of monocyte-associated genes. However, only a subset of these cells expressed M-CSF-R (CD115) and lysozyme mRNA, reminiscent of monocyte-related DC2 subset, which we discussed earlier in man and mouse. The authors also noted that the T-bet^−^ population was heterogeneous with respect to expression of C-type lectin receptors and consisted of a CLEC10A^−^ CLEC12A^−^, of a CLEC10A^−^ CLEC12A^+^ and of a CLEC10A^+^CLEC12A^+^ population.

Interestingly the T-bet^+^ cDC2 gene expression signature subset was neither detected in mouse nor in human blood (89). However, gene expression analysis of two samples from melanoma patients identified a cluster of myeloid cells that expressed T-bet target genes and additional signature genes like AREG and NR4A3. Also, in human spleen CD301^−^ DCs with transcriptomic similarities to murine T-bet^+^ DC2A cells were described.

Brown et al. (89) suggest that the human blood CD1c^+^ cDC2s might be analogous to the mouse T-bet^−^ CLEC10A^+^ DC2B, while in human blood there is no homologue of the mouse T-bet^+^ cDC2s. Clearly more studies, including functional tests, are required in order to collate these recent findings into a unified scheme and to align subsets across species.

### DC2 Subsets in the Mouse in Lung

For the lung of wild type mice, it was shown that there are CD14^+^ cells among a population of CD11b^+^CD24^+^CD64^−^ DC2s (90). Also, the DC2 cells were either Irf4^+^ or Irf4^−^ and in *Irf4* ^−/−^animals the CD24^+^ cells were decreased with a residual population remaining. Hence, one might speculate that the CD14^+^ cells represent an IRF4-independent population of mouse DC2 cells.

Recently, Bosteels et al. have shown that in the mouse lung CD26^+^CD172^+^ DC2s in response to type I interferon can up-regulate IRF8 and CD64 and acquire features of both DC1s and monocytes (91). This suggests that there is further complexity of dendritic cells in inflammation, and it stresses the necessity to carefully delineate DC subsets in disease settings, where such inflammatory DC2s increase. Analysis of ESAM and CLEC12A (CD371) in these cells, and their progenitors may help to elucidate the relationship to the two DC2 subsets described for the mouse (83, 84).

Taken together, also in the mouse lung, DC2s and their subsets can be readily detected.

## Concluding Remarks

There is a long-standing effort to dissect monocytes/macrophages and dendritic cells (3). This task becomes easier when the focus is set on bona fide DCs, i.e. the pDCs, CD1c^+^ DCs, and the CD141^+^ DCs in human blood. Still, there is concern that there might be overlap between the monocyte and DC lineage in some areas.

Regarding the CD1c^+^ DCs in blood, the open question then remains whether the CD14^+^ subset represents bona fide DCs or whether they belong to the monocyte lineage. The expression of markers like CD115 and S100A by these cells as well as their low antigen presenting capacity would support a monocyte nature.

**Figure 3:**
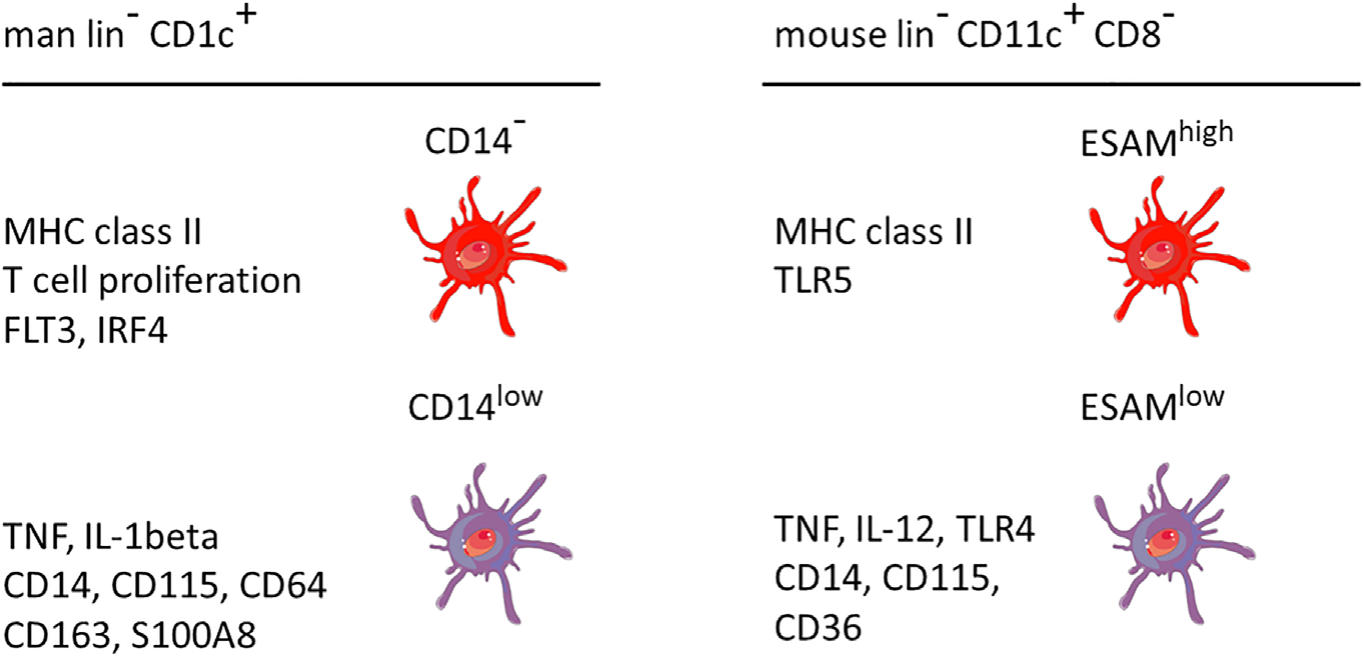
Properties of two main DC2 subsets in man and mouse. The markers are listed based on a higher expression in the respective subset compared to the other subset, i.e. the other subset can also be positive but at a lower level. This cartoon is restricted to the subset with monocyte features as compared to a subset covering the remaining DC2 cells. The latter has been reported to include up to three distinct populations as detailed in the text. lin^−^ = lineage negative. The human data are a summary of a series of studies compiled in **Figure 2**. The mouse data refer to Lewis et al., 2011, and Kasahara et al., 2012. The cellular images are provided and adapted from Servier Medical Art (smart.servier.com).

A comparison between man and mouse of the DC2 subsets without and with monocyte features is given in **Figure 3**. In both species the monocyte-related subset (green in **Figure 3**) is characterized by higher expression of cell surface molecules like CD115 and cytokines like TNF.

It remains to be resolved, whether these monocyte features may be explained by i) DC-lineage cells developing monocyte features, by ii) the cells being derived from mature classical monocytes similar to monocyte-derived DCs generated in GM-CSF cultures *in vitro* or iii) whether these cells are derived from a bone marrow precursor in common with monocytes.

Steps to be taken to resolve this issue are

- comparative single cell transcriptomics including CITE-seq approaches with a large set of prototypic monocytes/macrophages and dendritic cells from the same donor across different tissues,
- analysis of informative immune-deficiencies including knock-outs to study the mouse CD8^−^CD11c^+^CD11b^+^ ESAM^low^ DCs,
- development from hematopoietic progenitor cells with or without bar coding, and
- analysis of informative mutations in clonal hematopoiesis.

As mentioned earlier, a recent study has addressed some of these points and has looked into IRF8 immunodeficiencies and into *in vitro* development of DCs from progenitors. Here, differential transcription factor requirements for the CD14^+^ and the CD5^+^ subset of DC2s were apparent, and the CD1c^+^CD14^+^ DC2s showed co-segregation of with monocytes (63). While the issue of lineage assignment of the CD1c^+^CD14^+^ cells in human blood still needs to be resolved, it has become clear that there may be more than two subsets of DC2. Therefore, future work will have to address, in man and in the mouse, all subsets of DC2s and their role in homeostasis and in disease in order to arrive at an adequate nomenclature.

## Author Contributions

LZ-H conceived the project. LZ-H, MD, and DD drafted the paper. All authors contributed to the article and approved the submitted version.

## Funding

DD was partly supported by grants from the German Research Foundation [Deutsche Forschungsgemeinschaft (DFG)] (CRC1181-TPA7 and DU548/5-1), Agency national research (ANR)/German Research Foundation program (DU548/6-1) Bayresq.Net (Bavarian Ministry of Sciences), and Interdisziplinäres Zentrum für Klinische Forschung (IZKF-A80). LH was supported by Erlanger Leistungsbezogene Anschubfinanzierung und Nachwuchsförderung (ELAN) (DE-17-09-15-1-Heger). VB was supported by the Wellcome Trust grant 101155/Z13/Z.

## Conflict of Interest

The authors declare that the research was conducted in the absence of any commercial or financial relationships that could be construed as a potential conflict of interest.
